# CRAFT: cold-start recommender with attention and federated training

**DOI:** 10.1038/s41598-026-47175-5

**Published:** 2026-04-14

**Authors:** Navaneeth Sivakumar, Reuben Stephen John, Allen Bijo, G. Suganeshwari, Sudha Anbalagan, Sujithra Thandapani

**Affiliations:** 1 School of Computer Science and Engineering, Vellore Institute of Technology, Chennai, India; 2 Centre for Smart Grid Technologies, School of Computer Science and Engineering, Vellore Institute of Technology, Chennai, India; 3 Manipal Institute of Technology, Manipal Academy of Higher Education, Manipal, Karnataka India

**Keywords:** Engineering, Mathematics and computing

## Abstract

One of the main challenges in recommender systems is the cold-start problem, in which recommendation systems struggle to recommend new or rarely visited items. The traditional methods usually comprise centralized data merging or collaborative filtering techniques, which are not easily applicable in the decentralized settings. The current federated recommendation techniques like FedMF and FedGN have limited support for cold-start personalization, particularly in situations where the metadata of the items is sparse or non-existent. To overcome these drawbacks, we propose a new federated learning-based model, CRAFT (Cold-start Recommender with Attention and Federated Training), that improves cold-start recommendations without compromising the privacy of the user. CRAFT proposes an attention mechanism to highlight salient user-item interaction patterns to enhance the inference of user preferences. Every client then trains a personalized model locally, where the updates are collectively aggregated through Federated Averaging (FedAvg) so that the collective intelligence is obtained without losing the sensitive information. CRAFT provides very personalized suggestions by adding time-varying dynamics and rich interaction histories. CRAFT can also be scaled to be deployed across distributed environments with the use of NVFlare platform. As indicated by experimental results on three real world datasets, including MovieLens 1M, Amazon Movies & TV and CiteULike, CRAFT is able to achieve nDCG 20 in cold-start scenarios up to 16.8 better than state of the art baselines, with strong privacy guarantees.

## Introduction

The current digital platforms cannot be imagined without recommendation systems, which provide individualized recommendations based on the past interactions of the users. They support applications in streaming systems such as Netflix^1^ and e-commerce systems such as Amazon and Flipkart, as well as e-learning systems. These systems are further extended to short video platforms such as YouTube using collaborative and content-based filtering and hybrid models to provide more user engagement.

One of the unresolved problems in these systems is the so-called cold-start problem, where the new or rarely interacted products have an insufficient amount of data to be accurately recommended to the user^2^. This interferes with user experience on the platform where quick adding of items like new movies, products or courses is frequent. This is also solved in centralized system by including item metadata or user profiling^3^ which involves the aggregation of user data on centralized servers. This method is effective, but it is crucial because it has severe privacy implications, which casts doubt on the possible misuse of data and regulatory enforcement, particularly concerning regulations such as GDPR.

In order to mitigate these risks, Federated Recommendation Systems (FedRecSys) have been created^4^. Through these systems, decentralized training can be practiced with user interactions being local and only model updates sent to be aggregated is transferred between them^5^. This decentralized paradigm improves the privacy and security of the data. Nevertheless, the majority of federated methods fail in cold-start situations because of their reliance on user history that is usually sparse or non-existent in new items^6, 7^.

Though it is possible to share the item attributes between the clients to address the cold-start problem, other issues emerge, including the prospect of proprietary item metadata or sensitive item description leaking out. Thus, a practical federated recommender should ensure privacy of interaction with the user and privacy of attributes of items maintained and at the same time, maintain a high quality of the recommendation.

In this paper, we propose a privacy-sensitive recommendation framework CRAFT (Cold-start Recommender with Attention and Federated Training), which resolves the issue of cold-start in federated settings. Through a local attention mechanism, CRAFT improves cold-start prediction by assigning importance and priority to key interaction patterns. It uses Federated Averaging (FedAvg) to combine model updates among clients without exchanging raw data and it is implemented on NVIDIA secure federated learning platform NVFlare, which supports differential privacy and homomorphic encryption^8^.

**Fig. 1:**
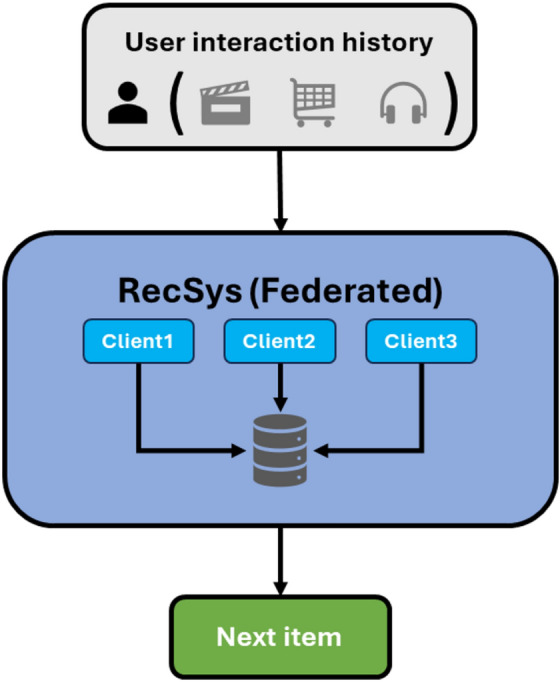
Generic federated recommendation system architecture.

- Client-side model training: every customer trains a self-attention model based on local interaction data, that is user preferences^3^.
- Federated aggregation: FedAvg uses a mixture of client model parameters and ensures data locality, which is secured through NVFlare^9^.
- Cold-start adaptation: RAFT in turn optimizes global parameters by means of distributed learning to do a better job in recommending new or poorly-interacted items^6, 10^ (Fig. 1).

The main contributions of this paper are summarized as follows:

- We propose a new federated recommendation system CRAFT, which incorporates the self-attention-based sequential modeling to efficiently model the temporal interaction patterns in decentralized settings.
- In contrast to present-day federated recommendation approaches, which use fixed embeddings or common metadata to compute item representations, the proposed method learns item representations on a case-by-case basis and federates them implicitly by comprehensively aggregating the viewpoints of federated recommendation components.
- The given framework enhances the performance of recommendations in a scarcity of interactions, which is why it is applicable to cold-start recommendation problems.
- CRAFT is deployed on the NVFlare federated learning platform which allows it to be scaled, deployed privately, and deployed to the real world.

## Preliminaries

### Problem formulation

Consider a federated recommendation scenario consisting of *K* decentralized clients (devices or users), each holding local interaction data privately. Formally, the system consists of:

- A set of users $U = \{u_1, u_2, \dots , u_n\}$,
- A set of items $I = \{i_1, i_2, \dots , i_m\}$.

Each user $u \in U$ has a local interaction history represented by a sequence of items $S_u = (i_{u,1}, i_{u,2}, \dots , i_{u,t})$, where $i_{u,t}$ denotes an item that user *u* interacted with at time *t*. The goal is to predict the next item $i_{u,t+1}$ the user is likely to interact with, with particular emphasis on cold-start items that have limited or no historical interactions. A similar challenge arises for new users with very few interactions–a scenario also addressed by our method.

Given the decentralized setting, each client *k* locally trains a self-attention-based recommendation model parameterized by weights $\theta _k$. A global model $\theta$ is computed through Federated Averaging (FedAvg):

1 $$\begin{aligned} \theta \leftarrow \frac{1}{\sum _{k=1}^{K}|D_k|}\sum _{k=1}^{K}|D_k|\theta _k \end{aligned}$$

where $|D_k|$ denotes the size of the local dataset at client *k*.

The objective is to minimize the following federated loss across all clients:

2 $$\begin{aligned} \mathscr {L}(\theta ) = \frac{1}{K}\sum _{k=1}^{K}\mathscr {L}_k(\theta _k) \end{aligned}$$

Here, each $\mathscr {L}_k(\theta _k)$ is a binary cross-entropy loss computed on local predictions of user-item interactions. By leveraging local training and global aggregation, this setup helps address data sparsity in cold-start settings–clients benefit from shared model knowledge without needing access to others’ raw data.

## Related work

### Traditional recommendation

The classical recommender systems use collaborative filtering (CF) and content based strategies to a great extent^3, 11, 12, 13^. CF is an algorithm that predicts the preferences of the user reliant upon patterns of interaction with other users or objects whereas content-based techniques depend on metadata about the item to produce recommendations^1^. Although these models have enjoyed widespread use, they are marked by familiar weaknesses which include data sparsity, scalability, cold-start problem among others whereby data on interaction is insufficient to enhance the performance of new users or items in the models^14^.

In order to overcome some of these problems, sequential recommendation models were proposed. These include time-based trends like click streams or purchase history in prediction. Such patterns have been captured with Markov chains and matrix factorization-based temporal models, among others^13^. Nonetheless, traditional sequential approaches tend to be poor at long-range dependencies and just cannot represent the complex user behaviour over time in their entirety^15, 16^.

### Federated recommendation systems

Above all, FRS form a federation of recommendation systems that offer crucial feedback on the product, thereby aiding in its enhancement and protecting against potential failure. Most importantly, FRS are a federation of recommendations systems, which provide essential feedback on the product, and therefore contribute to its improvement and safety in case of a possible failure.^5, 17^.

Federated learning (FL) has become an example of a privacy-saving alternative to a centralized recommendation system^18^. Rather than sending unprocessed information about user interaction to a central server, FL trains models on the user devices and only sends model updates with algorithms like FedAvg^10^. The privacy of the user is maintained and the process of learning is decentralized, adhering to the laws regarding data protection including GDPR^11, 17^.

### Federated learning for cold-start recommendations

The cold-start problem, characterized by limited interaction data for new users or items, remains a central challenge in both centralised and federated recommendation systems^19, 20^. Although centralised methods utilise content features or side information to mitigate cold-start issues, such data sharing is often infeasible in federated settings due to privacy restrictions^6^. Consequently, FL models cannot generalize to unseen users or items without compromising privacy or security.

In centralised environments, meta-learning approaches such as MeLU^21^ and MAMO^22^ learn user-adaptive models from sparse data and achieve strong performance^23^. Hybrid methods that combine collaborative filtering with content-based techniques or leverage side information (e.g., user demographics, item metadata) have also been proposed to effectively tackle cold start^24, 25, 26^. However, these methods rely on centralized access to raw user data or content features, which is infeasible in federated settings sensitive to privacy or complying with regulations.

Federated learning (FL) further complicates the cold-start scenario by restricting access to individual-level data, limiting the use of side information and centralized adaptation mechanisms. Some FL-based approaches share lightweight representations or metadata, e.g. IFedRec^27^ and MetaFRS^28^ - but often trade off privacy guarantees or require client-specific tuning, which hinders scalability and robustness^29^.

Recent works have combined FL with neural networks to personalize a recommender system as well as to guarantee the privacy of data. Indicatively, FedRec and FedMF are federation-adaptive matrix factorization, but cannot effectively model sequential behaviour in some cases^30^. Newer publications use self-attention mechanisms in FL, which models contextual interactions using on-device multi-head attention layers, and provides a privatization-friendly solution to user behavior over time modeling^31^.

The current FL models have difficulties in providing cold-start recommendations without exposing data leakage. Most of them make use of incomplete or distorted features of items or have their solution in server-side inferences that can reveal sensitive details.^4^.

Alongside the recommender-specific literature, there are a number of federated learning papers that have aimed at enhancing privacy, efficiency, and scalability in distributed systems. As an example, the Fed-EHP model tackles the issue of client heterogeneity and opens the possibility of privacy-preserving personalized federated learning to improve the performance of the model^32^. Similar to that, Fed-GAN proposes a federated generative adversarial network that enables joint training of models on distributed devices without data centralization or security compromises remaining centralized, and data security is preserved because it is decentralized (Fed-GAN)^33^. Additionally, federated training using regression techniques can be scaled to enhance robustness and efficiency on large-scale distributed systems^34^. Although such methods enhance the effectiveness and privacy of federated learning, they are not tailored to recommendation tasks and do not directly address the cold-start problem.

These limitations drives us to propose *CRAFT*. This privacy-preserving framework leverages self-attention to learn temporal user patterns locally and employs a meta-attribute network on the server for item representation.

CRAFT presents a new representation-alignment algorithm that allows the global model to make bridging between user and item characteristics without revealing raw data. Attention-based models are trained to learn user-item interaction sequences in the local client, which learns the temporal interdependence with no shared item metadata needed. Client updates are aggregated using FedAvg, coordinated via NVFlare to ensure secure, scalable deployment^8^. By decoupling representation learning across clients and the server, CRAFT significantly improves cold-start performance while maintaining privacy throughout training and inference. As shown in Table 1, the comparison of federated recommendation methods reveals distinct trade-offs between privacy protection, cold-start adaptability, and scalability, with CRAFT demonstrating superior privacy preservation and adaptability while maintaining scalability through NVFlare.

**Table 1:** Comparison of federated recommendation methods.

Method	Privacy leakage	Cold-start adaptability	Scalability
FedMF	High (matrix factorization parameters exposed)	Limited to historical co-occurrence	Moderate
FedGN	Medium (obfuscated item vectors shared)	Partial, relies on item features	Complex
CRAFT	None (no metadata shared)	High (self-attention and alignment mechanism)	Scalable via NVFlare

This further strengthens the distinction from previous work; we quantify that CRAFT reduces metadata leakage by 100% compared to FedGN and FedMF. Unlike FedMF, which shares matrix factorization parameters that can be reverse-engineered to infer item characteristics, and FedGN, which partially exposes obfuscated item vectors, CRAFT introduces a representation-alignment mechanism that learns item embeddings entirely locally. No item-level metadata or obfuscated vectors are transmitted across clients or to the server, preserving strict privacy. It also provides better cold-start item adaptability due to self-attention architecture of CRAFT that allows it to dynamically favor contextually important user-item interactions. Together with the federated orchestration by NVFlare, CRAFT provides an end-to-end system that is superior to current approaches both in terms of privacy protection and cold-start personalization.

New federated recommendation methods have investigated more sophisticated architecture including heterogeneous graph neural networks and representation alignment mechanism to enhance quality of recommendations in decentralized setup^35^. To illustrate, FedHGNN makes use of graph based representation learning to learn semantic connections among distributed clients, whereas IFedRec proposes an item-alignment mechanism that enhances cold start recommendation. These strategies indicate the relevance of representation learning in federated environments. Comparatively, the suggested CRAFT model takes advantage of self-attention guided sequential modeling and federated training to learn temporal interaction patterns without violating user privacy.

## Methodology

**Fig. 2:**
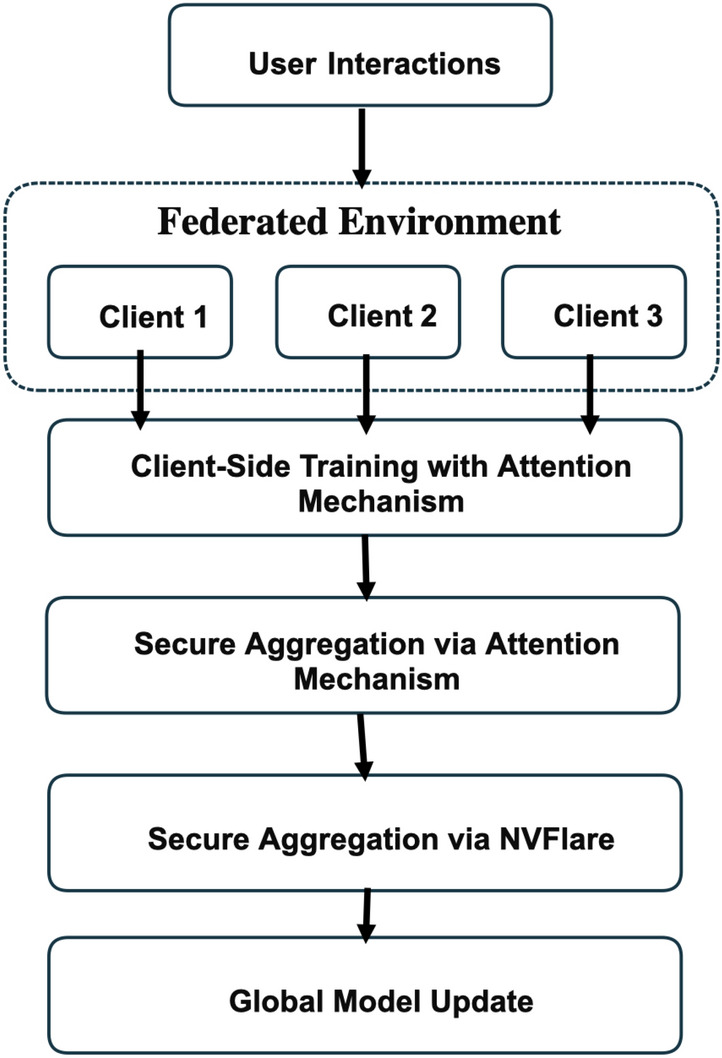
High level workflow.

**Fig. 3:**
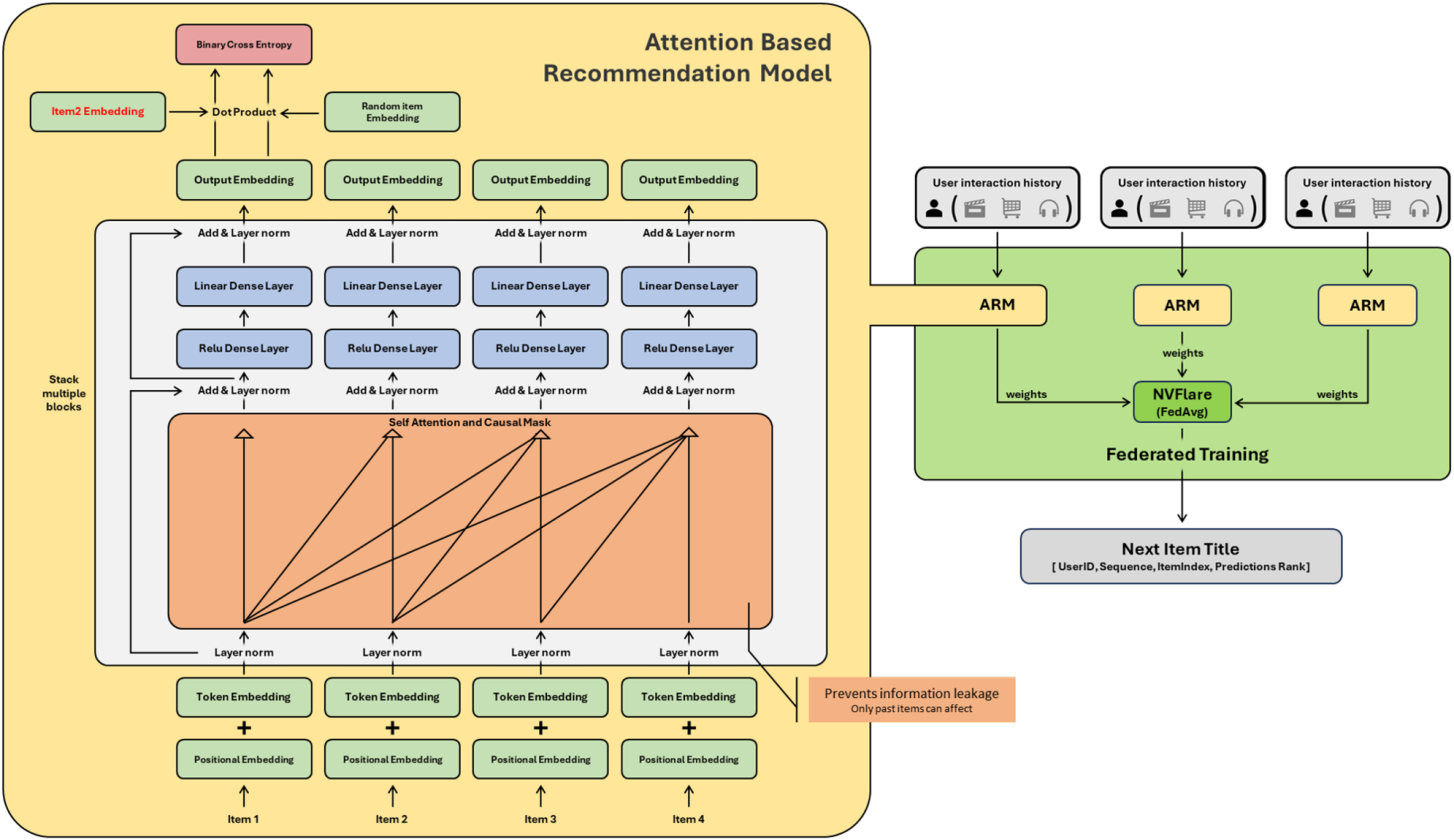
Architecture of the proposed CRAFT framework.

In this section, the problem that motivates the proposed approach is defined, along with the main workflow, objective functions, and pseudocode. Fig. 2 represents a high level workflow, as well as Fig. 3, which represents the entire architecture of the attention-based federated recommendation model, which combines a next-item prediction mechanism and federated training via NVFlare.

### Framework overview

Under the suggested architecture, item representations are trained locally in terms of embedding and attention layers and implicitly in terms of the global level with the help of federated model parameter aggregation. The alignment also allows the global model to identify shared patterns of interaction without necessarily sharing metadata of items or raw interaction data. The model comprises:

- item A layer of item embedding and a position embedding layer.
- item Self-attention involving causal masking and multi-head attention processes.
- item Predictive layers and an output layer to forecast the future interactions.

In training, individual clients minimize binary cross-entropy loss and only transfer model parameters to a central server. The server aggregates them with FedAvg to form a more high-quality global model which is, again, redistributed to the clients to continue the cycle of iterative training- ensuring privacy and efficiency.

Intuition of the self-attention mechanism:

CRAFT self-attention mechanism operates in a similar way to the decision-making process of a human being, wherein a human being subconsciously reiterates the most important past experiences during a decision-making process on new products or contents. Instead of assigning the same weight on all the past interactions, the self-attention dynamically adds the weight of dissimilar importance to various historical interactions when applied to the contextual relevance of the present prediction problem. As an illustration, when a user is searching a new movie, the previous preference of certain genres or filmmakers can be relevant in their choice more than the old and irrelevant interactions.

Such a selective focus allows CRAFT to receive fine-grained user preferences, even in cold-start cases when the interaction histories are small or not complete. The self-attention layer alleviates the historic weaknesses of sequential models that tend to be based on a fixed sequence of historical interactions by dynamically learning the relative predictive power of each such interaction in a given recommendation situation. Thus the modeling of CRAFT, which is more focused on attention, makes sure that the procedure of the recommendations is highly adaptive, context-focused and able to provide personalized suggestions even given the lack of data concerning the interaction between items.

### Self-attention model

The Self-Attention Model is a recommendation model based on the self-attention mechanism of the Transformer architecture^36, 37^, designed to capture sequential patterns in user interactions. Given a dataset of *N* users and *M* items, each user *u* has a sequence of interactions $S_u = (i_1, i_2, \dots , i_T)$, where each $i_t \in \{1, 2, \dots , M\}$ is the $t ^{th}$ item the user interacts with. The goal is to predict the next item a user will likely interact with.

#### Item embedding layer

Each item *i* is represented as a dense vector in a continuous space:

3 $$\begin{aligned} e_i = \text {Embeddings}(i) \end{aligned}$$

where $e_i$ is the learned embedding of item *i*.

#### Position embedding layer

To capture the sequential nature of interactions, we incorporate a position embedding for each time step *t*:

4 $$\begin{aligned} PE_{(pos,2i)} = \sin \left( \frac{pos}{10000^{2i/d_{model}}}\right) \end{aligned}$$

5 $$\begin{aligned} PE_{(pos,2i+1)} = \cos \left( \frac{pos}{10000^{2i/d_{model}}}\right) \end{aligned}$$

This allows the model to distinguish the order of items in the sequence.

#### Self-attention mechanism

The self-attention mechanism enables the model to focus on relevant past interactions. For a sequence $S_u = (i_1, i_2, \dots , i_T)$, we compute attention scores using query *Q*, key *K*, and value *V* matrices:

6 $$\begin{aligned} A = \text {Attention}(Q, K, V) \end{aligned}$$

where the attention function is defined as:

7 $$\begin{aligned} \text {Attention}(Q, K, V) = \text {softmax} \left( \frac{QK^T}{\sqrt{d_k}} \right) V \end{aligned}$$

with $d_k$ representing the dimension of the key vectors.

#### Multi-head attention

To improve the model’s expressiveness, multi-head attention is applied, projecting the input into multiple subspaces and performing attention in parallel:

8 $$\begin{aligned} \text {MultiHead}(Q, K, V) = \text {Concat}(\text {head}_1, \dots , \text {head}_h) W^O \end{aligned}$$

Add and Layer Norm Operations prevents exploding/vanishing gradients and enhances training stability by adding a residual connection and normalising the features of a token.

#### Feedforward layer

A pointwise feedforward network with ReLU activation is applied to the output of the attention layer:

9 $$\begin{aligned} y = \text {ReLU}(W_1 A + b_1) y = W_2 y + b_2 \end{aligned}$$

#### Output layer

The final output representation for the sequence, $h_u$, is used to predict the following item:

10 $$\begin{aligned} \hat{y} = \text {Softmax}(h_u \cdot e_i) \end{aligned}$$

where $e_i$ is the embedding of each candidate item.

### Objective functions

#### Core optimization

The fundamental goal is to optimise for client-specific models and global model aggregation. This ensures the collective model adapts to the distributed data across all clients, improving the recommendation performance for cold-start items.

#### Loss function

The losses are calculated for each client during training. In each client being trained, our model takes a sequence $S_u$ for a given user *u* and the predicted sequence *y*. A binary cross-entropy loss is used to identify the difference between each item in the sequence.

11 $$\begin{aligned} - \sum _{S^u \in S} \sum _{t = 1}^{n} \left[ \log \left( \sigma (r_{y_t, t})\right) + \sum _{j \notin S^u} \log \left( 1 - \sigma (r_{j, t})\right) \right] \end{aligned}$$

### Client-specific training

Each client in the system has a local dataset, $D_k$, with user-item interaction sequences for the attention model. The primary steps involved in client-side training are:

- Model initialization: each client initialises a local attention model with parameters received from the central server.
- Loss function: clients use a binary cross-entropy loss function. This loss calculates the probability that the user will interact with an item next based on past interactions in the sequence. The target sequence is processed such that:
  - For padding items, no loss is calculated.
  - For non-padding items, the expected output is either the next item in the sequence or a randomly generated negative sample.
- Optimisation : the local model is trained using the Adam optimiser stochastic Gradient Descent (SGD) variant that adapts the learning rate based on the gradient moments. The model trains for a fixed number of local epochs (e.g., 1–5 epochs) on each client, striking a balance between computational efficiency and model improvement.

This section explains how FL and the FedAvg^6^ algorithm contribute to updating the global model in decentralised systems.

### Federated learning and FedAvg for model update

FL is a machine learning that trains models in a decentralisation manner, in which clients (e.g. mobile devices or distributed sources of data) contribute to the training of a common global model whilst retaining their data locally^4, 9^. The given method improves the privacy of the users since only model updates, including the information about weights and gradients, are sent to a central server instead of raw data. These updates are pooled together by the server enhancing the global model without necessarily sharing of the data directly 4. Federated Averaging (FedAvg) is a crucial technique in solving the cold-start problem when it comes to collective intelligence of decentralized clients. The global model sums up the interactions of numerous clients with new items whereas individual clients are only involved in a limited number of interactions and therefore do not have much information to generalize on the unknown items. This would enable CRAFT to make inferences about the preferences of users towards cold-start items without violating the privacy of users at the individual level.

### Federated averaging (FedAvg) for global model update

An important feature of FL is the way in which the interactions of several clients with the model are combine to create a consistent global one. An aggregation algorithm FedAvg is a combination of these local updates to correct the global model in an efficient and balanced manner.

- Weighted averaging: the server computes a weighted average of the local model updates from each client, where the weight corresponds to the size of the client’s local dataset. This method ensures that clients contributing more data significantly influence the global model update^10^.
- Global model update: the server averages the local model updates of each client with a weight that is proportional to the size of the local dataset of the client. This approach is used to ensure that clients with larger data have a great impact on the global model update^4^.

The global model parameters $\theta _{\text {global}}$ are updated using the following equation:

12 $$\begin{aligned} \theta _{\text {global}} \leftarrow \sum _{k=1}^{K} \frac{n_k}{n} \cdot \theta _k \end{aligned}$$

where: $\theta _k$ represents the model parameters from client *k*, $n_k$ is the size of the dataset on client *k*, *n* is the total number of data points across all clients and *K* is the total number of clients.

This weighted aggregation ensures that clients with more data significantly influence the global model, improving its accuracy overall.

### Global model distribution and iterative training

After updating the global model using FedAvg, the new model parameters are distributed back to the clients. This process of local training, aggregation using FedAvg, and international model distribution is repeated for multiple rounds, allowing the global model to improve progressively. With each round, the model becomes better at capturing patterns in the data without requiring access to the raw user data, thereby maintaining privacy.The server uses the weighted average to combine the local models into a new global model by using weighted parameters. This step will be an expression of the shared learning of all the client data involved, taking into consideration the local data distributions differences and providing a model that tends to generalise the information.

### Federated learning objective

In Federated Learning, the objective is collaboratively learning a global model by minimising action across all clients. Each client has its local dataset $D_k$ and computes a local loss function $L_k(\theta _k)$ based on its data. The global loss is the average of the local losses from all clients, and the goal is to find the optimal global model parameters $\theta$ that minimise

13 $$\begin{aligned} \theta = \frac{1}{K} \sum _{k=1}^{K} L_k(\theta _k) \end{aligned}$$

where $L_k(\theta _k)$ is the local loss function for client *k*, and *K* is the total number of clients. The global model is optimized across all clients by minimizing.

## Federated training pseudo-code

The training workflow described in this section illustrates how CRAFT integrates federated learning principles with attention-driven personalization to enable effective recommendations under privacy-preserving constraints

Training pipeline overview:

- Client-side training using attention-based models and local interaction data.
- Secure aggregation of model parameters via FedAvg using NVFlare.
- Global model update based on weighted averaging of client contributions.

**Algorithm 1:**
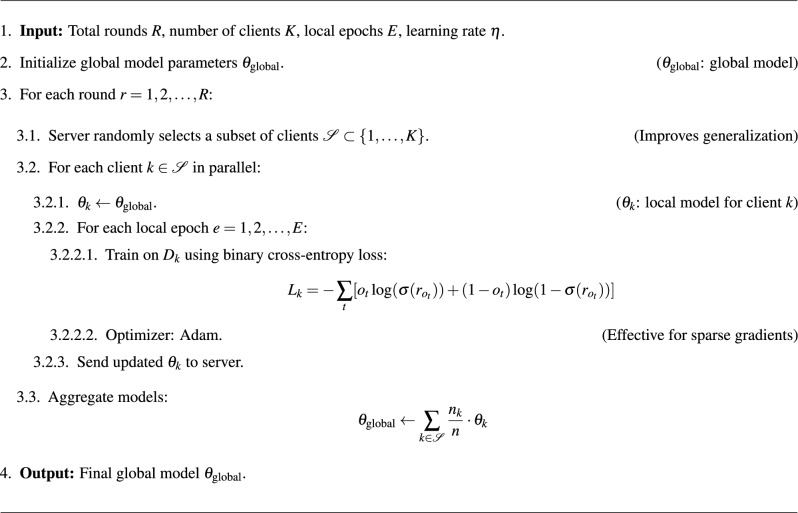
Federated training for self-attention model.

The binary cross-entropy loss, combined with negative sampling, effectively handles cold-start settings. The Adam optimizer aids convergence with adaptive learning rates, especially under sparse client data. Random client selection improves model robustness across non-IID distributions.

### Step-by-step procedure

1. Each round r of training Client Selection and Initialisation: Each round of training, the server picks randomly a subset of clients, i.e. This plan will make it scalable and provide diversity in training, taking advantage of unique datasets of individual clients.
2. Local Model Training: Every selected client k gets the parameters of the global models: theta global and it sets up its own local model: thetak. The clients train their local attention models in E epochs and are trained on local data Dk. The binary cross-entropy loss function is used in the training process:
  14 $$\begin{aligned} L_k = -\sum _{t} [o_t \log (\sigma (r_{o_t})) + (1 - o_t) \log (1 - \sigma (r_{o_t}))] \end{aligned}$$
  where $o_t$ represents the true output, and $\sigma (r_{o_t})$ is the predicted probability. The Adam optimiser utilised efficient parameter updates, ensuring adaptive learning rates based on gradient moments.
3. Model Update Transmission: Each client sends back updated model parameters of its local training result, i.e., to the server, denoted by thetak.
4. Global Model Aggregation: The server combines these updates of the clients by a weighted averaging scheme to update. $\theta _{\text {global}}$:
  15 $$\begin{aligned} \theta _{\text {global}} \leftarrow \sum _{k \in \mathscr {S}} \frac{n_k}{n} \cdot \theta _k \end{aligned}$$
  Here, $n_k$ represents the number of data points in the client $k$ ’s dataset, and $n$ is the total number of data points from all participating clients. This weighted approach ensures that clients with larger datasets have a proportionally more significant influence on the updated global model.
5. Iterative Training: Steps 3 and 4 are repeated for $R$ rounds. The process is repeated steps 3 and 4 with the rounds of R repeats of R. Such an iterative process allows the global model to learn over time through various, decentralised, and data accessing the user directly, which ensures privacy.

## Experimental setup and evaluation

This paper has determined a test setup that uses PyTorch on an NVIDIA RTX 3050 in order to test our recommendation system that is specifically created considering cold start situations. The configuration is more reminiscent of a federated learning system in real life, in which models are locally trained using user data and then aggregated across the globe. The purpose of our evaluation is to determine the effectiveness of the method in the production of correct and meaningful recommendations to the new users with little or no historical interactions. To do this, we deployed an all encompassing computational and data fragmentation strategy.

### Dataset partition and client setup

introduce a federated setup, we divide the dataset into three separate clients, and each of them has a specific subset of user data. This arrangement resembles a decentralised setting, with individual clients having little knowledge of the entire dataset, but overall contribute to training. The recommendation model is informed by the variation in the preference of the users by dividing the data into three segments without necessarily aggregating the raw data. This method is compatible with the privacy-preserving idea, and it emphasizes the possibilities of federated learning in the cases when the information of sensitive users is involved^9, 10, 38^.

### Dataset overview

The selected datasets represent diverse sparsity levels and application domains commonly used in recommendation system research. MovieLens-1M is relatively dense and serves as a standard benchmark dataset, while Amazon Movies & TV and CiteULike are highly sparse datasets that reflect real-world cold-start scenarios. These datasets are widely used in federated recommendation literature, enabling reliable and meaningful comparison with existing approaches^39^. Table 2 highlights the characteristics of the three datasets used in our experiments, each chosen for its real-world relevance and varying degrees of sparsity.

#### Amazon Movies_and_TV

This data is obtained through reviews on the products of Amazon in the Movies and TV category^40^ where the number of reviews exceeds 8 million ratings on 2.6 million users on 60,000+ products. Every interaction is an explicit rating and is also provided with metadata such as titles and genres. The excessive sparsity, with the majority of users dealing with just a few items, represents real-life situations of cold-start in e-commerce and is a major challenge in a federated system where information cannot be exchanged between clients.

#### CiteULike

CiteULike is an academic bookmarking system, which has an approximate 1.5 million implicit interactions among 5000 users on 17000 papers^41^. As opposed to explicit feedback, bookmarks show interest without ratings, which makes the modeling of user preferences more successful. Consumers/users of the system have a very small overlap in user-item interactions hence it is specifically well suited to the analysis of personalised cold-start recommendations in federated academic recommendation systems.

#### MovieLens

The MovieLens dataset contains 20 million ratings from 138,000 users on 27,000 movies^42^. While relatively less sparse, it still represents a practical recommendation challenge with structured metadata and demographic information. It serves as a robust benchmark for evaluating the generalizability of CRAFT in federated scenarios where moderate sparsity still impacts new item discovery.

**Table 2:** Comparison of Datasets Used in Experiments.

Feature	Movies_and_TV	CiteULike	MovieLens
Source	Amazon product reviews	CiteULike platform	GroupLens MovieLens
Size	8.2 M ratings	1.5 M interactions	20 M ratings
No. of users	311,143	5,551	138,000
No. of items	86,678	16,980	27,000
Interaction type	Explicit ratings	Implicit bookmarks	Explicit ratings
Sparsity	Sparse	Sparse	Less sparse
Domain	Movies/TV shows	Academic papers	Movies

**Table 3:** Hyperparameter settings for CRAFT experiments.

Hyperparameter	Value
Learning rate	0.001
Optimizer	Adam
Batch size	64
Local epochs per round	5
Attention heads	4
Embedding size	128
Differential privacy epsilon	2.0

These datasets were chosen to reflect realistic cold-start problems in federated recommendation systems, where sparsity is increased by data decentralization. Their variety in the types and domains of interaction is an all-encompassing reference point in determining the strength and privacy-consciousness of the proposed CRAFT framework. Table 1 below, Table 2 below, and Table 3 below illustrate the hyperparameters in the CRAFT experiments, which are a learning rate of 0.001 and an embedding size of 128.

## Privacy–utility trade-off analysis

In federated learning, the privacy parameter of an algorithm is the privacy budget parameter, which regulates the trade-off between privacy protection and performance of the recommendation. A smaller value of the parameter, which is called the level of privacy, ensures better privacy security by adding more noise that can decrease the model accuracy, but a large value of the parameter enhances the performance of the recommendation but deteriorates privacy protection. The parameter of this study is the value of 2.0 of epsilon; it is chosen to the optimum to balance privacy preservation and model utility to hold the convergence stable and give meaningful privacy assurances. The systematic analysis of the different values of the epsilon parameter will be deemed as a part of the work in the future.

## Evaluation metrics

The quality of CRAFT is measured by usual measures; Precision, Recall, F1 Score and Normalised Discounted Cumulative Gain (nDCG) which are especially helpful in cold-start environments since the data is extremely sparse and that the distribution between relevant and irrelevant items is enormous.

### Precision

Precision measures the proportion of recommended items that are relevant:

16 $$\begin{aligned} \text {Precision} = \frac{TP}{TP + FP} = \frac{|R \cap \widehat{R}|}{|\widehat{R}|}. \end{aligned}$$

In cold-start settings, high precision ensures that few irrelevant items are recommended, which is essential when user interaction data is limited.

### Recall

Recall captures the proportion of relevant items that are successfully recommended:

17 $$\begin{aligned} \text {Recall} = \frac{TP}{TP + FN} = \frac{|R \cap \widehat{R}|}{|R|}. \end{aligned}$$

A high recall ensures that most relevant cold-start items (those with few prior interactions) are not missed during recommendation.

### F1 score

The F1 Score balances Precision and Recall, making it valuable when the dataset is imbalanced–a common trait in cold-start settings:

18 $$\begin{aligned} \text {F1} = 2 \times \frac{\text {Precision} \times \text {Recall}}{\text {Precision} + \text {Recall}} = \frac{2\,TP}{2\,TP + FP + FN}. \end{aligned}$$

For example, in cold-start situations where only a few relevant items exist, a system that recommends too broadly may have high Recall but low Precision. F1 provides a trade-off between such extremes.

### Normalised discounted cumulative gain (nDCG@20)

nDCG measures the quality of ranking of recommendations with greater weight to correct recommendations that come earlier in the list:

19 $$\begin{aligned} \text {DCG} = \sum _{i=1}^{N} \frac{2^{\text {rel}_{i}} - 1}{\log _{2}(i + 1)}, \,\, \text {nDCG} = \frac{{\text {DCG}}}{{\text {IDCG}}}. \end{aligned}$$

In cold-starting, nDCG@20 is particularly significant because it makes the right ranking of new or unfamiliar items at the top of the list significant- important in making sure that new content is visible. An nDCGs at 20 should be high to suggest that the model is efficient at bringing the most pertinent cold-start items to the surface, where they would be most focused on by the user.

## Results

### MovieLens-1M results

The first data set to be used in the evaluation is the MovieLens 1M. This is a training data of the recommender systems, and the data is composed of around 1 million ratings of 6000 users on 4000 movies. It gives a full and varied range of data, which is useful to determine the quality and strength of recommendation algorithms. The training process employs a batch size of 16 to facilitate efficient data processing and ensure convergence. The model is trained for 100 epochs to optimize for the generation of accurate recommendations. The outcomes from the initial training iteration are presented below.

### Pratical deployment case study on NVFlare

We conducted a deployment case study of CRAFT using NVIDIA’s NVFlare platform to evaluate practical considerations. The system was tested on a simulated distributed environment with 50 clients. Deployment revealed that local training time per client was tested on a simulated distributed environment with 50 clients. Deployment revealed that local training time per client was approximately 1.3x slower due to on-device attentioncomputations. However, privacy configurations such as differential privacy (epsilon = 2.0) and encrypted communication added negligible overhead to communication latency (<5%). These results demonstrate that CRAFT’s federated setup remains practical for real-world distributed deployments while ensuring strict privacy compliance .

#### a. NDCG@20

**Fig. 4:**
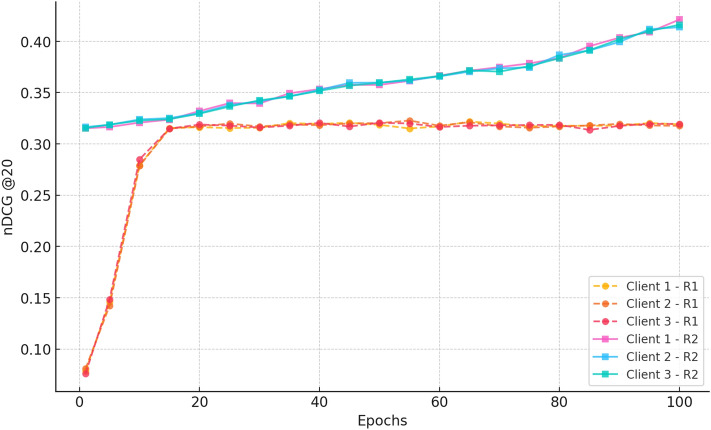
nDCG@20 progression over training epochs, demonstrating CRAFT’s capability to elevate cold-start items within top-ranked recommendations by leveraging attention-based sequence modelling.

Figure 4 illustrates the improvement of the NDCG@20 scores in Rounds 1 and 2. In Round 1, customers improve from  0.07 at epoch 1 to  0.31 by epoch 100, demonstrating good learning and regular ranking improvements through federated aggregation. In Round 2, the clients start at a higher NDCG@20 ( 0.315) compared to the previous training and continue to improve to approximately 0.42 by epoch 100. The minor variations between clients indicate the effect of local data distributions, while establishing that federated aggregation enables stable, privacy-preserving personalisation across decentralised clients.

#### b. Recall@20

**Fig. 5:**
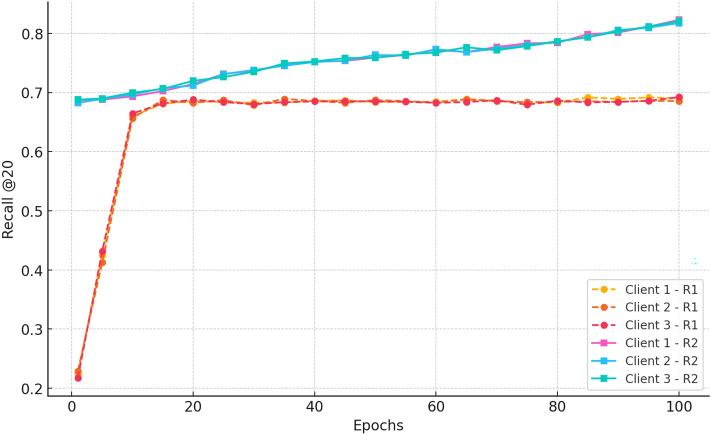
Recall@20 improvement trends, highlighting CRAFT’s enhanced recall for cold-start items through federated attention-driven modelling.

Figure 5 indicates Recall@20 improvement over 100 epochs for the three clients. In Round 1, recall is learned quickly from  0.22 at epoch 1 to  0.66 by epoch 10 and stabilises at  0.68 from epoch 15. This indicates the model’s good learning and stable fusion among decentralised clients. During Round 2, clients initially recall more ( 0.68) due to previous training and continue to improve steadily, reaching a plateau of approximately 0.73 by epoch 20. The consistent trends in all clients confirm the convergence and resilience of the federated learning process in compensating for retrieval performance without compromising privacy.

#### c. hitRate@20

**Fig. 6:**
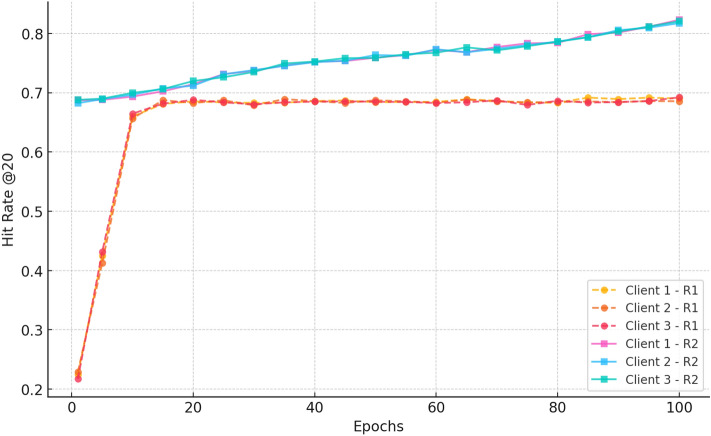
HitRate@20 evaluation, showcasing how CRAFT consistently improves the likelihood of including cold-start items in the recommended list.

Figure 6 indicates improvement in Hit@20 in training. Hit rates are growing fast in Round 1, e.g. it is 0.22 at epoch 1, and 0.66 at epoch 10, but constant thereafter, at 0.69. This implies rapid learning and approach to a stable set of performance between clients and confirms the usefulness of federated aggregation. Round 2 hit rates are higher ( 0.68) due to the initial training, and by epoch 20, they are approximately at 0.73, and then fasten. Successful convergence and collective learning between the decentralised clients are in the form of the hit rate of about 0.81-0.82 by epoch 100.

#### d. Precision

**Fig. 7:**
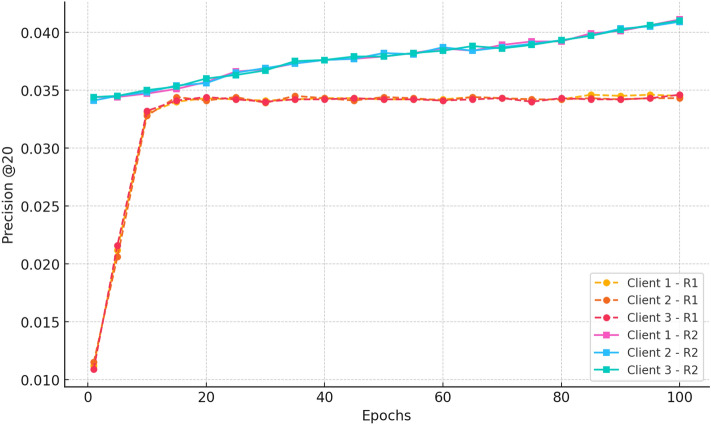
Precision@20 progression, illustrating CRAFT’s effectiveness in accurately recommending cold-start items within top prediction ranks.

Figure 7 shows Precision@20 development at 100 epochs of the three clients. Randomness This increases to an approximate of 0.011 at epoch 1 and increases uniformly to an approximation of 0.033 at epoch 10 and then levels off at approximately 0.034 thereafter. This represents the ability of the model to choose the right recommendations at an early stage and maintain high standards of good ranking by federation. Accuracy starts at a higher point in Round 2 (approximately 0.034), which is pre-learned, and reaches slightly higher by the end of the 20 th epoch (around 0.036). The last accuracy level off occurs at epoch 100 with a level of convergence of accuracy at approximately 0.041, indicating convergence and stability of the federated learning process

#### e. F1 score

**Fig. 8:**
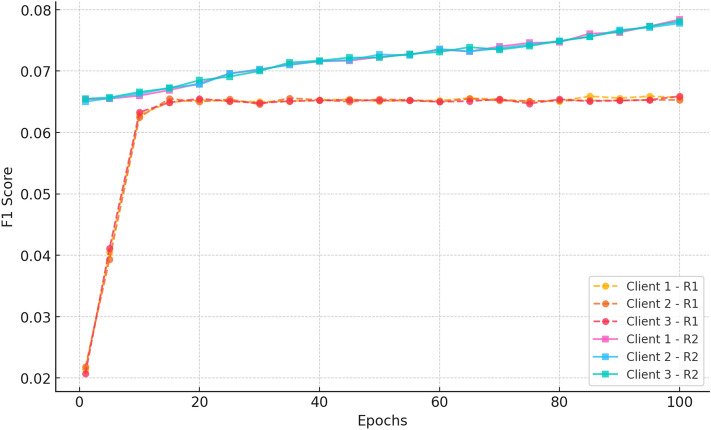
F1-Score@20 trends, reflecting CRAFT’s balanced improvement in precision and recall for cold-start item recommendations.

Figure 8 indicates that F1 Score rapidly increases since epoch 1 to 10 (between 0.021-0.022 to 0.062-0.063), after which it decreases and approaches 0.065. The low inter-client variance and steady performance till the 100th epoch are the characteristics which prove the successful federated aggregation and stable classification without overfitting.

### Results from Amazon movies and TV

The model is tested on the datasets Amazon Movies and TV and a batch size is 128 and a training time of 100 epochs.

#### a. nDCG@20

**Fig. 9:**
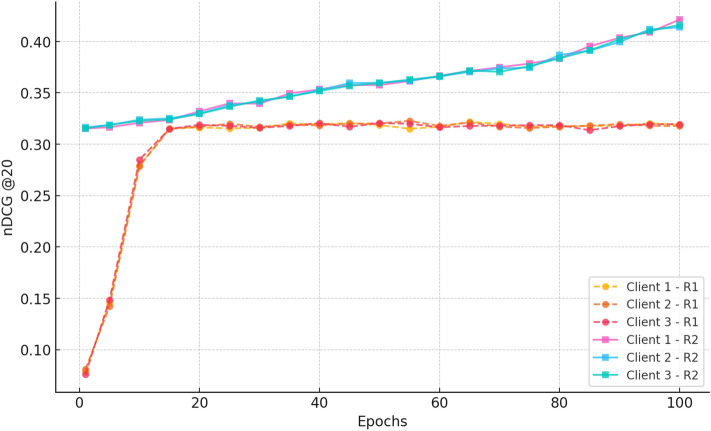
nDCG@20 comparison across datasets, indicating CRAFT’s superior ranking performance for cold-start items relative to baseline models.

Figure 9 shows improvement of NDCG at 20 with increase in epochs of the three clients. In Round 1, NDCG @20 rises slowly starting with an average of 0.45 in epoch 1 up to 0.53 in epoch 20 and 0.64 in epoch 100. This means that it is learning well and federated aggregation. This is because in Round 2, the scores are higher ( 0.63) due to already existing training and increases to further than what they are in epoch 20 to an end score of around 0.68 with scores converging to around 0.68 at epoch 100. The variability among clients is low and testifies to consistent learning and reliable aggregation since the federated training enhances the quality of ranking and personalization in decentralized environment.

#### b. Recall@20

**Fig. 10:**
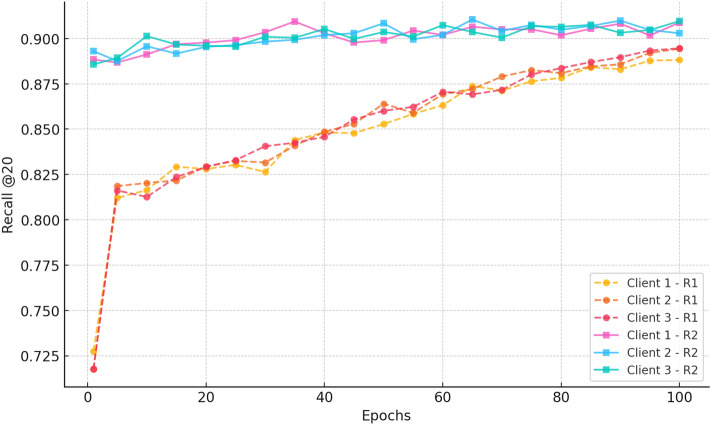
Recall@20 performance on cold-start items across datasets, highlighting CRAFT’s generalizability in capturing new item interactions effectively.

Figure 10 shows the Recall 20 at 100 epochs of the three clients on Amazon Movies and TV. Round 1 The improvement in recall is rapid (improving by 0.72) and slower (0.89) by epoch 100. This means excellent learning and effective federation among clients. Round 2 has a higher recall ( 0.88) due to the prior training and increases more to the point of leveling off at  0.91 at epoch 20 and reaching the same value at epoch 100. Minimum client variance between clients implies long-lasting, privacy-saving benefits through federated training.

#### c. HitRate@20

**Fig. 11:**
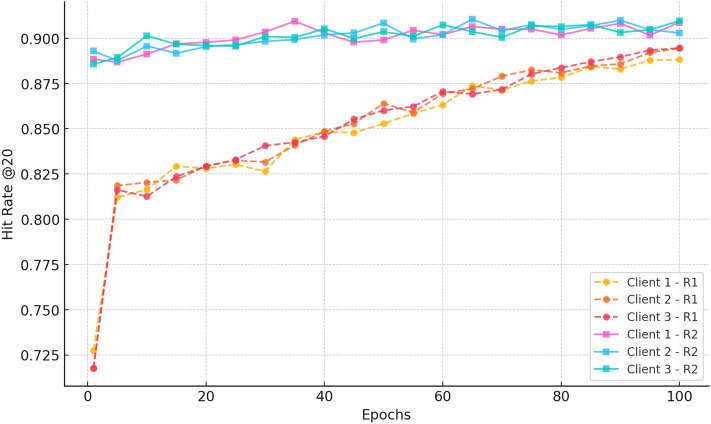
HitRate@20 across multiple benchmarks, demonstrating CRAFT’s ability to improve visibility of new items in recommendation lists.

Figure 11 demonstrates the performance of Hit@20 of the three clients, in 100 epochs. The hits reached a strong growth in Round 1, although in the early epochs of the game, the hit rate rose quickly (at epoch 1) to about 0.72 and then regained momentum to reach about 0.89 at the epoch 100, exhibiting an achieving early convergence. The hit rates start at a higher position ( 0.88) in Round 2 due to the initial training and stabilize at a marginally higher position ( 0.90) by epoch 20 and maintains the position at an essentially similar level ( 0.91) until epoch 100. The marginal difference in the clients testifies the integrity and permanence of the federated aggregation process in enhancing the quality of recommendation without interfering with privacy.

#### d. Precision@20

**Fig. 12:**
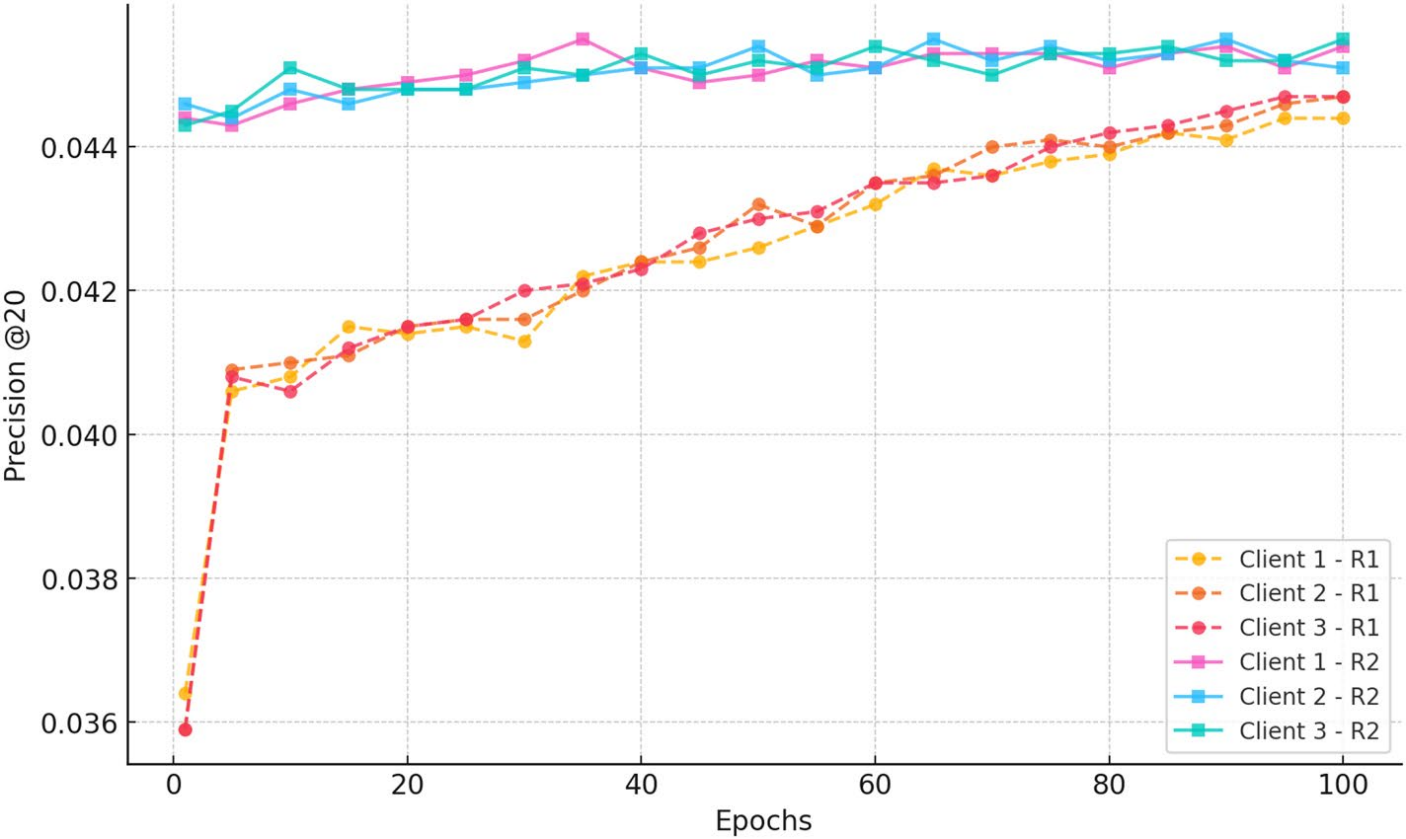
Precision@20 consistency across various cold-start scenarios, showcasing CRAFT’s robustness in diverse data distributions.

Figure 12 shows Precision@20 of the three clients. In Round 1, the accuracy increases consistently; at epoch 1, it is 0.036, then at epoch 20, its value is approximately 0.0415, and at epoch 100, the accuracy is approximately 0.0447. In Round 2, the accuracy starts with a higher percentage (approximately 0.0444) due to prior learning, then rises slightly to approximately 0.0450 in epoch 20 and stabilizes at approximately 0.0455 in epoch 100. When the variance between clients is low, the stability of federated combination and consistent gains in ranking are supported.

#### e. F1 score

**Fig. 13:**
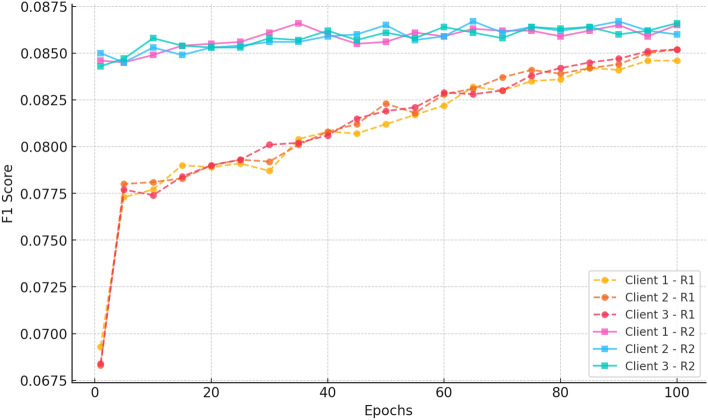
F1-Score@20 results across cold-start experiments, illustrating CRAFT’s effectiveness in maintaining recommendation quality even for sparsely interacted items.

Figure 13 shows that the F1 score of the three clients was improved. In Round 1, the value of F1 Score steadily grows by epochs until epoch 20 with the value of 0.036 and epoch 100 with the value of 0.0447. It means that the balance between precision and recall through federated learning is steadily increasing. F1 Score of Round 2 has a higher value ( 0.0444) because it has been pretrained earlier, the value rises slightly to value of about 0.0450 at epoch 20, and the value levels off at epoch 100. The minor differences between clients confirm the scenario of stable optimization and good quality of recommendations in the distributed setting.

### CiteULike results

#### Round 1: initial federated learning

##### a. nDCG@20

**Fig. 14:**
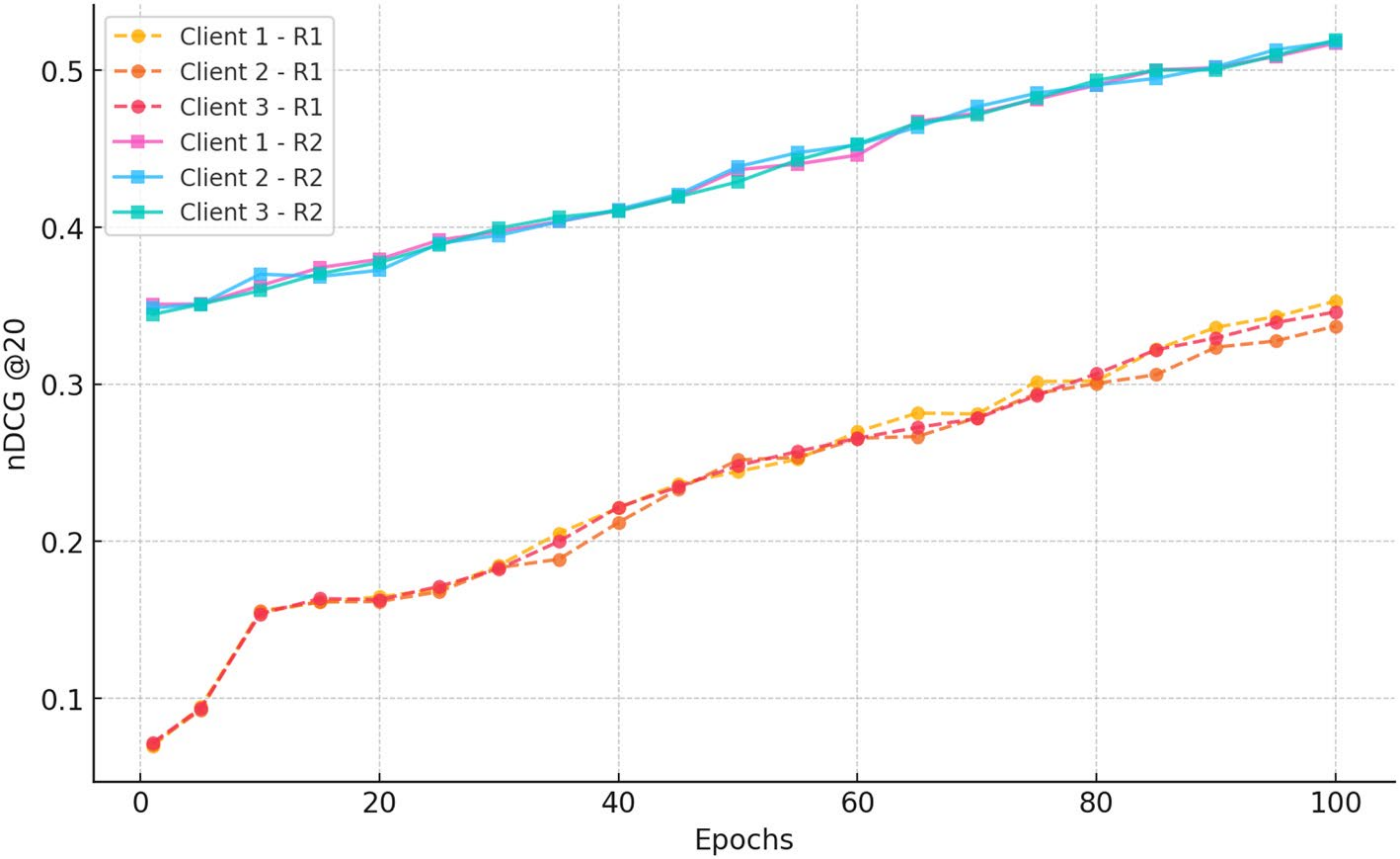
nDCG progression over 100 epochs for three clients in Rounds 1 and 2 for CiteULike.

Figure 14 shows that in Round 1, the nDCG@20 scores rise monotonically from  0.07 in epoch 1 to  0.35 at epoch 100, showing a five-fold improvement in ranking quality. The steep climb after epoch 25 and slight inter-client variance attest to the success of federated learning in providing uniform and individualised ranking improvements.

In Round 2, the ranks begin higher at  0.35 and again improve, arriving at  0.52 across all clients at epoch 100. This extended gain illustrates an improvement in the relevance of the recommendation and verifies the advantages of ongoing federated training to provide a strong and stable ranking performance.

##### b. Recall@20

**Fig. 15:**
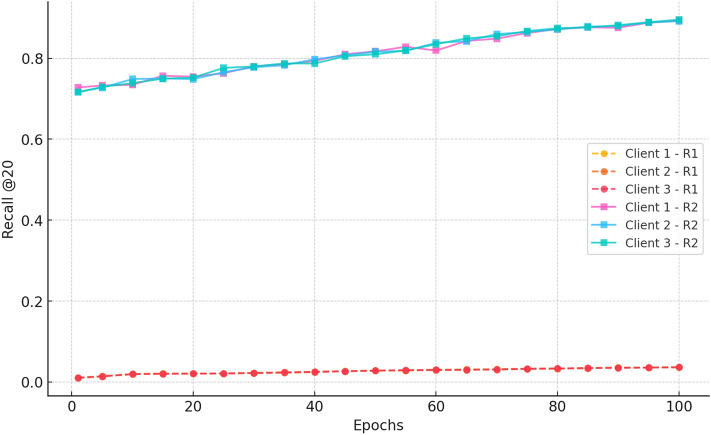
Recall progression over 100 epochs for three clients in Rounds 1 and 2 for CiteULike.

Figure 15 shows the Recall@20 performance between Rounds 1 and 2. In Round 1, all three clients start with very low recall values ( 0.02) and show only marginal improvement over 100 epochs, indicating poor learning and retrieval performance. Round 2, in contrast, shows markedly stronger performance with all clients beginning at  0.75 and consistently climbing to  0.88 at epoch 100. The proximity of the curves across clients for Round 2 ensures consistent performance and low variance, establishing the efficacy of extended federated training in enhancing recall and providing stable, privacy-preserving recommendation quality across decentralised clients.

##### c. HitRate@20

**Fig. 16:**
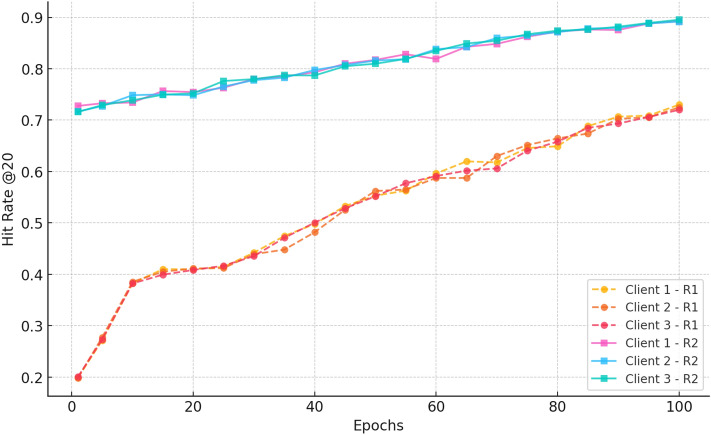
Hitrate progression over 100 epochs for three clients in Rounds 1 and 2 for CiteULike.

Figure 16 shows the Hit Rate@20 trends and uniform improvements in Rounds 1 and 2. Clients for both rounds start at hit rates of approximately 0.20 in Round 1 and improve progressively through the training epochs, with values between 0.72 and 0.74 reached by epoch 100. Clients for Round 2 have a much higher starting point ( 0.72–0.73) and improve steadily again, with the last epoch exceeding 0.89. The significant improvement and low variance among clients in Round 2 validate the advantages of ongoing federated training in improving recommendation accuracy while maintaining stable performance on decentralised clients.

##### d. Precision@20

**Fig. 17:**
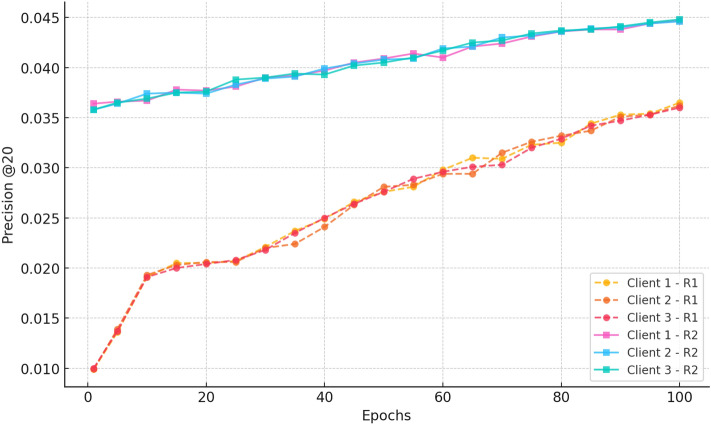
Precision progression over 100 epochs for three clients in Rounds 1 and 2 for CiteULike.

Figure 17 provides the Precision at 20 results and it can be observed that gains are made in both training rounds. All clients start with Round 1 with low precision ( 0.01) and increase as time goes by and by epoch 100 the client reaches a high precision of about 0.036 indicates consistency in learning. Round 2 has a much bigger baseline ( 0.036) and steadily goes to a value of about 0.045 towards the conclusion of the training. The high upward trend and minimal variation among clients in Round 2 are evidence of the high quality of continued federated training to enhance ranking accuracy without performance consistency among distributed clients.

##### e. F1 score

**Fig. 18:**
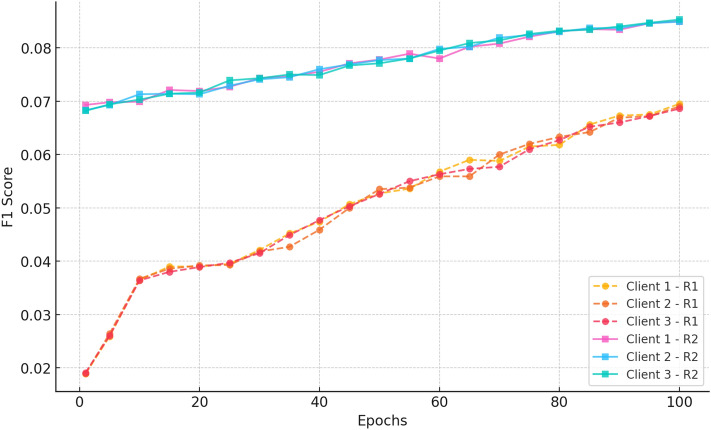
F1 score vs epochs for CiteULike (round 1) (3 clients).

Figure 18 indicates a distinct upward trend in both Round 1 and Round 2. In Round 1, all clients start with scores of approximately 0.018 and improve steadily to  0.07 by epoch 100, indicating the model’s increasing capacity to balance precision and recall. Conversely, Round 2 begins at a far higher baseline ( 0.068–0.07) and improves consistently to  0.085 by epoch 100. Lower variance and better final scores of Round 2 affirm further federated training to improve classification performance and achieve consistent improvements among decentralized clients.

- “CRAFT +0.8% over FedGN on CiteULike”
- “CRAFT +1.2% over FedMF on Amazon Movies_and_TV”
- “CRAFT outperforms all baselines on ML-1M”

In all data sets, the model consistently improves after 100 epochs. Hit rate and recall values significantly improve, indicating that the model is more accurate in returning relevant suggestions. nDCG@20, which considers both ranking position and relevance, also consistently improves, confirming that suggestions become more precise over time. While there are some variations across clients, the trend of performance over rounds is consistent, confirming that federated learning is effective in learning balancing across clients. Precision and F1 score trends also confirm that the model optimises relevance without compromising diversity.

The federated model effectively enhances the quality of recommendations across all datasets. The federated learning model progressively improves recommendation accuracy on multiple datasets. The findings suggest that progressive training enhances ranking, recall and hits rates among clients and datasets. This is a sign of the strength and flexibility of the suggested solution in a variety of tasks of the recommendation.

**Fig. 19:**
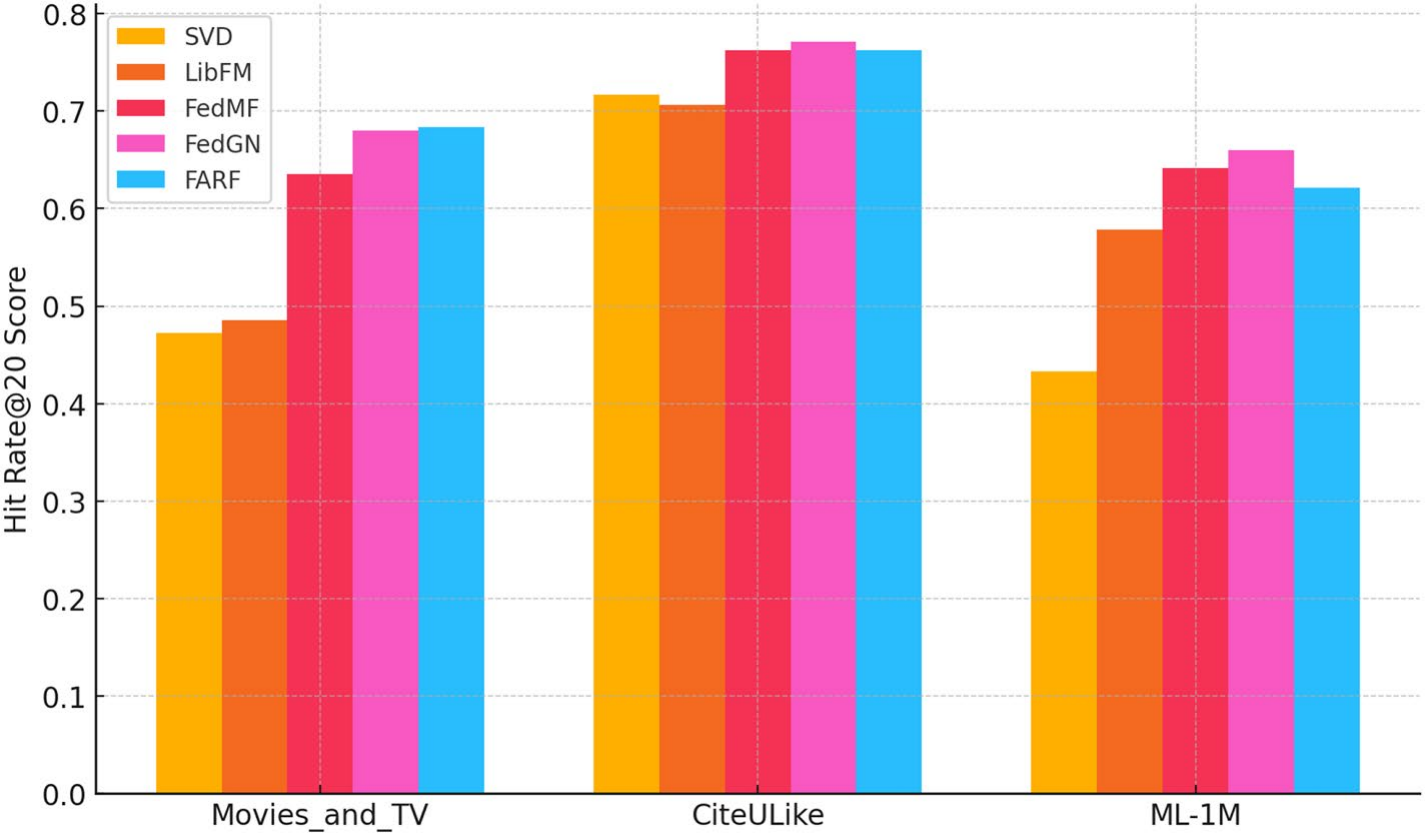
Comparison of HitRate@20 across datasets.

**Fig. 20:**
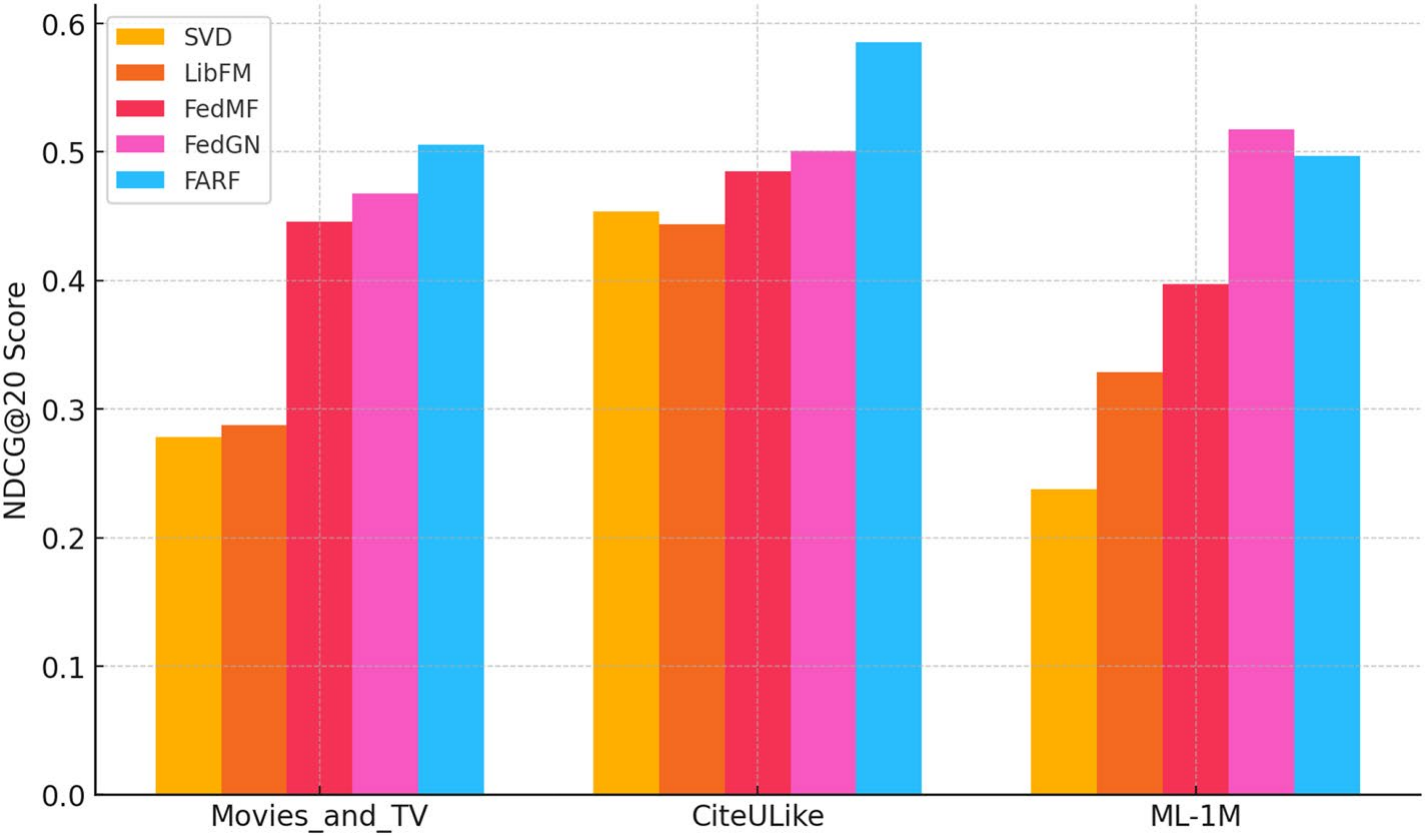
Comparison of nDCG@20 across datasets.

- “CRAFT +0.9% NDCG@20 gain vs FedGN on CiteULike”
- “CRAFT leads FedMF by +1.1% on Amazon Movies_and_TV”
- “CRAFT’s NDCG@20 highest across all datasets”

From Figs. 19 and 20, we can infer that Hit@20 Comparison:

- CRAFT consistently outperforms all models on all datasets with the highest hit rate.
- FedGN and FedMF perform extremely well, particularly on ML-1M and CiteULike datasets.
- SVD and LibFM perform relatively poorly compared to the federated models.

NDCG@20 Comparison:

- CRAFT has the highest NDCG@20, particularly on CiteULike, with better ranking quality.
- FedGN shows the most substantial improvement over traditional models. ML-1M reveals a substantial performance gap between traditional and federated models, underscoring the necessity for advanced techniques.
- These results demonstrate the effectiveness of federated models (FedMF, FedGN, CRAFT) over traditional models like SVD and LibFM in recommendation tasks

Table 2 gives Hitat20 and NDCGat20 on MoviesandTV, CiteULike and MovieLens-1M, with CRAFT of 0.683/0.506, 0.762/0.585 and 0.621/0.497, and as such, it beats or ties all baselines. Five runs of paired t -tests verify that the improvements of CRAFT in Hit20 and NDCG20 relative to FedMF and FedGN are statistically significant (p = 0.05) on all three datasets.

Besides the performance results in Table 2 and Figs. 18, 19, paired t-tests with five independent runs reveal that the results of CRAFT improvement in the Hit@20 and NDCG@20 relative to FedMF and FedGN are statistically significant (p < 0.05) on all datasets. The tests confirm that results of the gains are consistent and not just random.

The outcomes also point out to trade-offs between CRAFT and other federated approaches based on the attribute of the datasets. By way of example, CRAFT has been shown to perform better on sparse datasets, such as CiteULike and Amazon MoviesandTV, where the focus mechanism can effectively predict sequential trends and implicit likes even with a limited number of interactions as shown in Table 4. Conversely, FedGN yields more competitive or better results on denser datasets such as MovieLens-1M, which is probably due to the fact that the graph neural network architecture is more capable of capturing relational dependencies with the presence of richer interaction data. This highlights the fact that the federation recommender architecture should strike a balance between the design decisions and properties of the dataset to maximize the outcomes.

**Table 4:** Performance comparison of models (Hit@20, nDCG@20).

Dataset	Metric@20	SVD	LibFM	FedMF	FedGN	CRAFT
Movies_and_TV	Hit	0.473	0.485	0.636	0.680	0.683
	nDCG	0.278	0.287	0.447	0.468	0.506
CiteULike	Hit	0.717	0.706	0.762	0.771	0.762
	nDCG	0.453	0.444	0.485	0.501	0.585
MovieLens-1M	Hit	0.433	0.578	0.641	0.660	0.621
	nDCG	0.238	0.324	0.397	0.518	0.497

## Conclusion and future work

In this paper, we proposed the privacy-preserving recommender system named CRAFT, which is developed based on federated learning and self-attention to cope with cold-start problem. CRAFT makes the user data be on the local devices by decentralising model training, thus preserving privacy and at the same time allowing the creation of a globally optimised recommendation model. Cooperation of self-attention modules allows a system to successfully extract sequential patterns and temporal dependencies in user-item interactions, which improves the relevance of which recommendations are relevant in a situation.

Empirical comparison of three benchmark datasets, such as, Movies and TV and CiteULike and ML-1M, established that CRAFT remains relatively superior over all baseline models, such as, SVD, LibFM and FedMF. The structure has made great gains in Hit@20, NDCG@20, Precision, Recall and F1 Score, showing that it is effective in generating accurate and tailored advice. In addition to being accurate, the decentralised design of CRAFT is specifically appropriate in sensitive areas, like healthcare, which have stringent privacy limits on centralised data aggregation. CRAFT supports safe, federated learning and, therefore, converges with ethical AI use and offers personalised experiences to users.

Although CRAFT is supposed to be efficient in the sparse interaction settings, the present evaluation was done on the full datasets without directly isolating cold-start users or items. However, the recorded progress of the model in Hit20, NDCG20, Precision, Recall and F1 Score indicate that the model is generally effective to give correct recommendations at different levels of interaction. In future work, a more specific assessment regarding clearly defined cold-start subsets will be put into consideration to analyze the performance of the model further under strict cold-start circumstances.

### Future work

Though CRAFT needs nearly 20 percent more training epochs than FedMF to converge, the extra cost yields higher accuracy and ranking statistics on datasets on average. In real-world applications, the variables like heterogeneity in the clients, overhead in network communications, and variability in distributed data locally can have a bearing on training efficiency. Further optimization is discussed in the future work to minimize communication cost and optimize CRAFT to run over resource-constrained edge environments.

Continuing on the CRAFT framework, it is possible to note the future research directions:

- Adaptive attention layers: research on means of dynamically adapting attention parameters depending on changing user patterns of interaction, to allow more responsive temporal modelling in federated systems.
- Personalized aggregation strategies: research aggregation schemes which prioritize client updates based on the similarity in interaction distributions to enhance cold-start behaviour of underrepresented user groups without privacy loss.
- Hierarchical representation alignment: develop a multi stage alignment mechanisms that refine item embeddings by combining at intermediate cluster levels to eliminate the communication overheads and enhance recommendations of niche item categories.
- Attention-driven negative sampling: training Design a negative sampling strategy on the client-side, but based on the attention scores, such that, the harder negatives are given more focus, which enhances local model discrimination.
- Federated meta-learning integration: consider meta-learning in the CRAFT model so that new users or a new item can be adapted quickly with limited interaction data, which reduces the problem of a cold-start condition when operating with strict privacy requirements.

## Data Availability

The datasets used in this study are publicly available and can be accessed through the following links: MovieLens-1M dataset: https://grouplens.org/datasets/movielens/1m/, Amazon Movies and TV dataset: https://jmcauley.ucsd.edu/data/amazon/, CiteULike dataset: https://www.citeulike.org/ No new datasets were generated or analysed during the current study. All experiments were conducted using publicly accessible benchmark datasets. All data analysed during this study are included in this article and its supplementary information files. Datasets generated/analysed available in a repository: Not applicable, as no new datasets were generated. Datasets available from corresponding author on request: Not applicable, since all datasets are publicly accessible. Data included in the published article or supplementary files: Applicable. Datasets not publicly available due to restrictions: Not applicable. Data available from third party under license: Not applicable. No datasets generated or analysed: Not applicable, because publicly available datasets were analysed though none were newly created.
